# An Experimental and Numerical Analysis of the Compression of Bimetallic Cylinders

**DOI:** 10.3390/ma12244094

**Published:** 2019-12-07

**Authors:** Ana María Camacho, Álvaro Rodríguez-Prieto, José Manuel Herrero, Ana María Aragón, Claudio Bernal, Cinta Lorenzo-Martin, Ángel Yanguas-Gil, Paulo A. F. Martins

**Affiliations:** 1 Department of Manufacturing Engineering, Universidad Nacional de Educación a Distancia (UNED), 28040 Madrid, Spain; alvaro.rodriguez@invi.uned.es (Á.R.-P.); jherrero74@alumno.uned.es (J.M.H.); amaragon@invi.uned.es (A.M.A.); cbernal@ind.uned.es (C.B.); 2 Applied Materials Division, Argonne National Laboratory, 9700 Cass Ave, Lemont, IL 60439, USA; lorenzo-martin@anl.gov (C.L.-M.); ayg@anl.gov (Á.Y.-G.); 3 Instituto de Engenharia Mecânica, Instituto Superior Técnico, Universidade de Lisboa, Av. Rovisco Pais, 1049-001 Lisboa, Portugal; pmartins@tecnico.ulisboa.pt

**Keywords:** metal forming, bi-metallic, cylinders, compression, finite elements, experimentation, microscopy

## Abstract

This paper investigates the upsetting of bimetallic cylinders with an aluminum alloy center and a brass ring. The influence of the center-ring shape factor and type of assembly fit (interference and clearance), and the effect of friction on the compression force and ductile damage are comprehensively analyzed by means of a combined numerical-experimental approach. Results showed that the higher the shape factor, the lower the forces required, whereas the effect of friction is especially important for cylinders with the lowest shape factors. The type of assembly fit does not influence the compression force. The accumulated ductile damage in the compression of bimetallic cylinders is higher than in single-material cylinders, and the higher the shape factor, the lower the damage for the same amount of stroke. The highest values of damaged were found to occur at the middle plane, and typically in the ring. Results also showed that an interference fit was more favorable for preventing fracture of the ring than a clearance fit. Microstructural analysis by scanning electron microscopy revealed a good agreement with the finite element predicted distribution of ductile damage.

## 1. Introduction

In recent years, there has been a considerable growth in the use of multi-material components due to their advantages over single-material components regarding the possibility of tailoring physical properties, improving stiffness and strength, reducing overall weight, and saving the number of parts and the assembly costs in mechanical systems made of multiple components. Reductions in weight can, for example, be achieved through the combination of materials with lower densities than the original ones. Significant cost savings in electric power systems used in modern hybrid and electric vehicles can also be obtained by combining materials with different electrical and thermal conductivities. Improvements in the surface integrity of components exposed to extreme conditions are also of special interest; namely, in applications subjected to high friction contacts (enhancing the tribological properties) or high corrosion environments, such as those existing in marine and chemical industries [1]. In fact, the range of potential applications of multi-material components is so wide that they can also be found in the production of high denomination coins for security and aesthetic reasons.

The interest in multi-material components is scientifically and socially recognized by its inclusion in different work programs of the EU Horizon 2020, in which the manufacturing of multi-materials by additive manufacturing (for research, transport, customized goods, or biomaterials), and the combination of commercial materials into multi-material components for industrial applications [2] were selected as key research topics. The importance of additive manufacturing is confirmed by the growing number of publications in the field, which are focused on both directed energy deposition (DED) and powder bed fusion (PBF) [3] based techniques. Laser engineering net shaping (LENS) [4,5,6] is a powder DED based process; laser metal deposition (LMD) [7] is a wire DED based process; and selective laser melting (SLM) [8] is one of the most promising PBF processes for fabricating multi-material components.

Still, there are limitations in the use of additive manufacturing that are similar to those found in the fabrication of multi-material components by welding (e.g., friction stir welding, and laser and explosive welding) [5]. In fact, joining of dissimilar materials suffers from the risk of formation of brittle intermetallic metallurgical structures, and thermal heating-cooling cycles give rise to residual stresses, distortions, and geometric inaccuracies [3,9]. Table 1 summarizes the main problems associated with the production of multi-material components by additive manufacturing, welding, and forming.

As seen in Table 1, metal forming successfully overcomes most of the difficulties that are found in the production of multi-material components by additive manufacturing and welding. The main problems are due to formability issues and to the risk of delamination because thermal effects do not play a role in the cold metal forming based process that is considered in this paper.

Despite this, formability studies on multi-material components made from commercial materials by means of metal forming are not very widespread in literature. Studies are mostly limited to bimetallic components made of two different metallic alloys, such as the publications on the extrusion of bi-metallic components [10,11,12] and on the combination of forming and joining to produce bimetallic bearing bushings [13].

Coin minting of bimetallic disks is probably the most well-known application in the field [14,15] and the technology was recently thrown to a higher level of complexity by the development of new bi-material collection coins with a polymer composite center and a metallic ring to generate innovative aesthetics and incorporate advanced holographic security features [16]. Finite element modelling was utilized to investigate the influence of the initial clearance between the polymer center and the metallic ring on the mechanics of coin minting and performance of the resulting force fit joint.

Other researchers like Essa et al. [17] discussed the possibility of producing bimetallic components or prefroms by upsetting, after concluding that some geometries with a good interfacial contact between the center and the ring can be successfully employed as preforms for further processing. A similar conclusion was made by Misirly et al. [18] after analyzing the open die forging of bimetallic cylinders with steel rings and brass and pure copper centers, and observing that pure copper prevents the formation of cavities at the center-ring interfaces.

A recently publish work by Cetintav et al. [1] on the compression of trimetallic cylinders with aluminum centers and steel, copper, and brass rings, focused on the improvements in mechanical properties and weight reduction that result from the utilization of multi-material components.

More recently, Wernicke et al. [19] developed a new type of hybrid gear made from aluminum and steel to obtain significant weight reductions and locally adapted mechanical properties without the need of performing subsequent heat-treatment processes.

In the meantime, there have also been other investigations in the field aimed at analyzing the deformation mechanics and predicting the compression forces in multi-material components. This is the case of Plancak et al. [20], who developed two special purpose analytical models to calculate the compression force and validated their predictions against experimental tests performed on bimetallic cylinders with centers and rings made from different commercial steels. These models were later improved by Gisbert et al. [21] to include shear friction.

Under these circumstances, this paper aims to analyze the formability of bimetallic cylindrical billets produced by compression by means of a numerical and experimental based investigation. Compression forces and accumulation of ductile damage were analyzed by means of a work plan including different shape factors and two assembly fits between the center and the ring (interference and clearance). Scanning electron microscopy (SEM) observations were included to identify the major defects and to correlate the location of these defects with the finite element predicted distribution of ductile damage after compression.

The problems of delamination included in Table 1 will not be addressed because these are mainly found when the compression forces are not applied perpendicular to the contact surfaces between the different materials to be joined, as in case of extrusion and rolling [17]. This is not the case in the present investigation.

## 2. Materials and Methods

### 2.1. Materials and Experimental Work Plan

**Figure 1:**
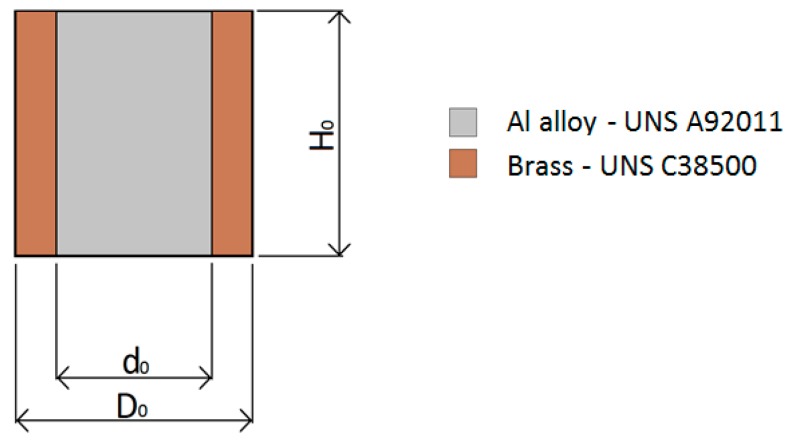
Bimetallic cylindrical test samples and notation utilized in the paper.

The bimetallic cylindrical test samples utilized in the investigation have an aluminum alloy UNS A92011 center and a brass UNS C38500 ring (Figure 1).

The aluminum center and the brass rings were machined from commercial rods with 12 and 15 mm diameters, respectively. Both materials were utilized in their as-supplied conditions and their chemical compositions are listed in Table 2.

The physical and mechanical properties of both materials are included in Table 3.

The density of brass is three times higher than that of the aluminum alloy but its beta metallurgical phase, which is very appropriate for applications with extreme contact pressures, limits its ductility in cold forming. The overall rigidity of brass is also higher than that of the aluminum alloy because the latter has a smaller yield stress and a smaller ultimate tensile strength (UTS), meaning that it requires less energy to be plastically deformed. The experimental work plan is summarized in Table 4 and made use of cylindrical test samples with different height to diameter ratios, H_0_/d_0_ (previously designated as the “shape factor”) and two different types of assembly fit. The assembly fit (P1i) corresponds to test samples in which the center was mounted into the ring with interference. For this purpose, the center was pushed into the ring using the universal testing machine that was also used in the compression tests. The assembly fit (P2i) corresponds to test samples in which the center was mounted into ring with a clearance of 0.1 mm in order to ensure easy sliding between the two parts.

**Figure 2:**
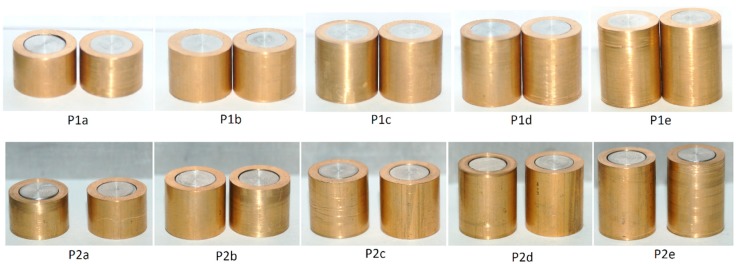
Bimetallic cylindrical test samples before compression. Notation in accordance with Table 4.

Figure 2 shows the bi-metallic cylindrical test samples (notation according to Table 4) before compression. Two samples were prepared for each testing condition.

### 2.2. Equipment and Experimental Procedure

The compression of the bimetallic cylindrical test samples was performed in a universal testing machine Hoytom HM-100kN (Hoytom HM-100kN, Hoytom, S.L., Leioa, Spain) with control software Howin 32 RS (version 3.11, Hoytom, S.L., Leioa, Spain). A precision cut-off machine Mecatome P100 (Mecatome P100, PRESI, Brié et Angonnes, France) was utilized to prepare the test samples for analysis and micrographic observation after compression.

The experimental procedure consisted of the following steps:

1. Before compression, the samples and the compression die platens were properly cleaned with ethanol.
2. The samples were then placed in the center of the lower die platen.
3. Compression was performed for each sample. Repeatability of the testing conditions (ram speed, pre-contact force and end of testing) was ensured by the control software of the universal testing machine. A pre-contact force of 50 N was utilized, after which the ram moved at a constant speed of 1 mm/s until reaching a compression force of 90 kN. The tests were performed at room temperature under dry, lubricated conditions.
4. After testing, the samples were cut along their axial cross-sectional plane with the precision cut-off machine.

### 2.3. Finite Element Modeling

**Figure 3:**
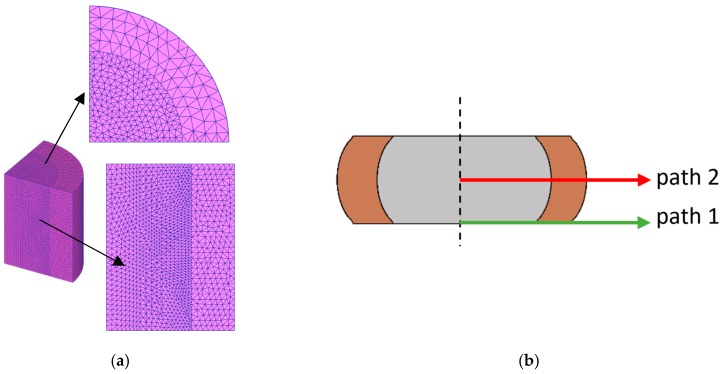
Finite element modelling of the compression of bimetallic cylinders: (**a**) Detail of the initial mesh; (**b**) Identification of the two paths (path 1 in green and path 2 in red) that will be later utilized in the presentation to analyze ductile damage.

Finite element simulations were carried out with the commercial finite element computer program DEFORM 3D. The compression die platens were modelled as rigid objects and the bimetallic cylinders were modelled as an assembly between two plastically deformable objects (center and ring). The center and ring were discretized by means of approximately 11,000 tetrahedral elements. The detail of the initial finite element meshes is provided in Figure 3a.

**Figure 4:**
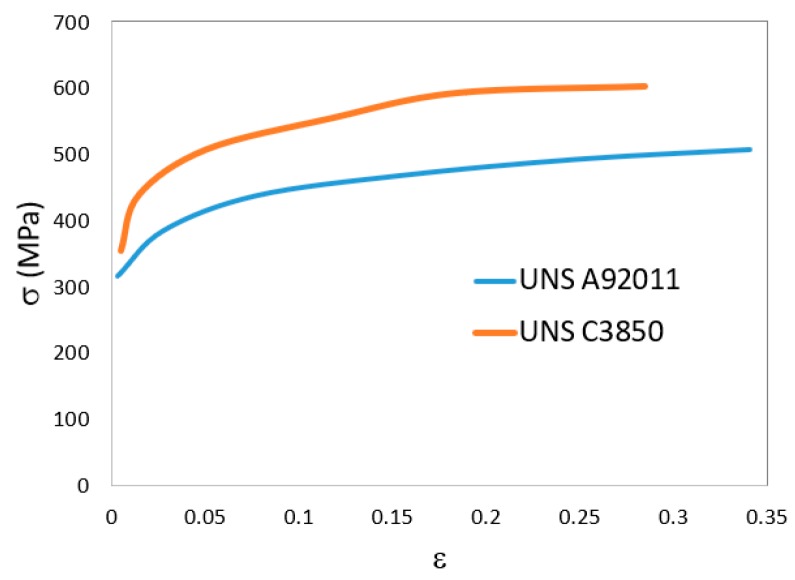
Flow curves of the aluminum alloy UNS A92011 and brass UNS C3850.

The center and ring materials (aluminum alloy UNS A92011 and brass UNS C38500) were assumed to be isotropic and their flow curves (true stress–true strain curves) are disclosed in Figure 4.

Friction was modelled by means of the law of constant friction. As explained by Essa et al. [17], it is not possible to accurately define the frictional conditions prevailing at the center-ring contact interface. But previous research in multi-material upsetting [1,17,18], also lead to the conclusion that variations of the friction factor in the range of 0 to 0.5 do not influence the overall deformation of multi-material components.

Therefore, taking into consideration a previous study performed by the authors [24], which points to the same above-mentioned conclusion, it was decided to use a friction factor equal to 0.08 along the center-ring contact interface. The same study was utilized to define a value of 0.12 at the contact interfaces between the specimen and the upper and lower die platens.

The accumulation of ductile damage *D* was modelled by means of the Cockcroft–Latham criterion [25]. According to this criterion, fracture is supposed to occur when the accumulated ductile damage reaches a critical value $D_{crit}$, for a given temperature and strain rate loading condition

(1) $$D_{crit}=\int_{0}^{{\bar{\varepsilon }}_{f}}\sigma^{*}d\bar{\varepsilon },$$

where σ* is the maximum principal stress, $\bar{\varepsilon }$ is the equivalent strain, and ${\bar{\varepsilon }}_{f}$ is the equivalent strain at fracture.

The ductile damage distributions included in this paper are based on a normalized version of the Cockcroft–Latham criterion (1),

(2) $$C=\int_{0}^{\bar{\varepsilon }}\frac{\sigma^{*}}{\bar{\sigma }}d\bar{\varepsilon },$$

where $\bar{\sigma }\;$ is the effective stress. The values of strain and stress were calculated at the centre of each tetrahedral element, and therefore, the values of the normalized accumulated ductile damage C were also accumulated at the centre of the elements.

Damage distribution along the two paths shown in Figure 3b were calculated during post-processing of results. Path 1 was taken at the contact interface between the deformed cylinders and lower die platen, and path 2 was taken from the middle plane of the cylinders after compression.

### 2.4. Scanning Electron Microscopy (SEM)

Microstructural observation and analysis of the test samples with interference (P1a to P1e) were carried out with a high-resolution scanning electron microscope of the Center for Nanoscale Materials (CNM) at Argonne National Laboratory. The equipment utilized was a Hitachi S-4700-II (Hitachi, Krefeld, Germany), with an electron dispersive spectroscopic (EDS) detector, Bruker XFlash 6160 (Bruker, Billerica, MA, USA).

P1a to P1e samples were microstructurally characterized because damage was observed but fracture did not occur. This approach allows one to assess the formability of this multi-material sample prepared with interference, so more useful information was obtained from the defects found in the microstructural characterization.

The results obtained from these observations were compared with the finite element predictions of accumulated ductile damage. Further validation of the finite element computations was performed by comparing the numerical and experimental force-displacement evolutions. This is displayed in the following section.

## 3. Results and Discussion

### 3.1. Compression Forces

**Figure 5:**
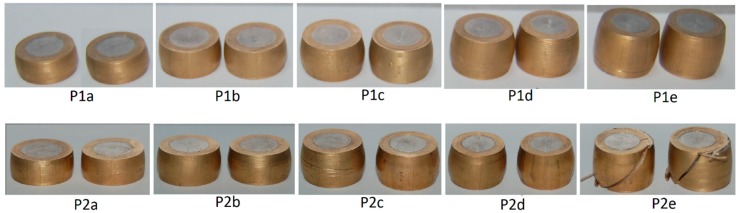
Bimetallic cylindrical test samples after compression. Failure by cracking is observed in sample P2e (Table 4).

Figure 5 shows the bimetallic cylindrical test samples after compression. As seen, the influence of the assembly fit only provides visible differences for the samples with a shape factor H_0_/d_0_ = 2 because of cracking in the samples where the center was mounted in the ring with clearance.

**Figure 6:**
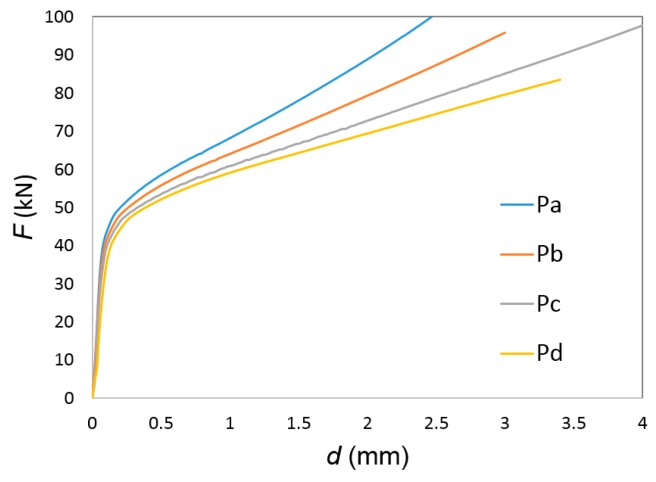
Finite element predicted evolution of the force with displacement for the compression of bimetallic cylindrical test samples with different shape factors H_0_/d_0_ (Pa: 1.00, Pb: 1.25, Pc: 1.50, Pd: 1.75).

The lack of visible differences in the other test samples with smaller shape factors H_0_/d_0_ was further confirmed by the finite element predicted evolution of force with displacement shown in Figure 6. In fact, the force-displacement evolution is only sensitive to the shape factors H_0_/d_0,_ as in case of single-material (solid) cylinders—the higher the shape factor, the lower the compression forces. In other words, there is no influence of the type of assembly fit on the force-displacement evolution for the test samples with shape factors H_0_/d_0_ = 1, 1.25, 1.5, and 1.75.

**Figure 7:**
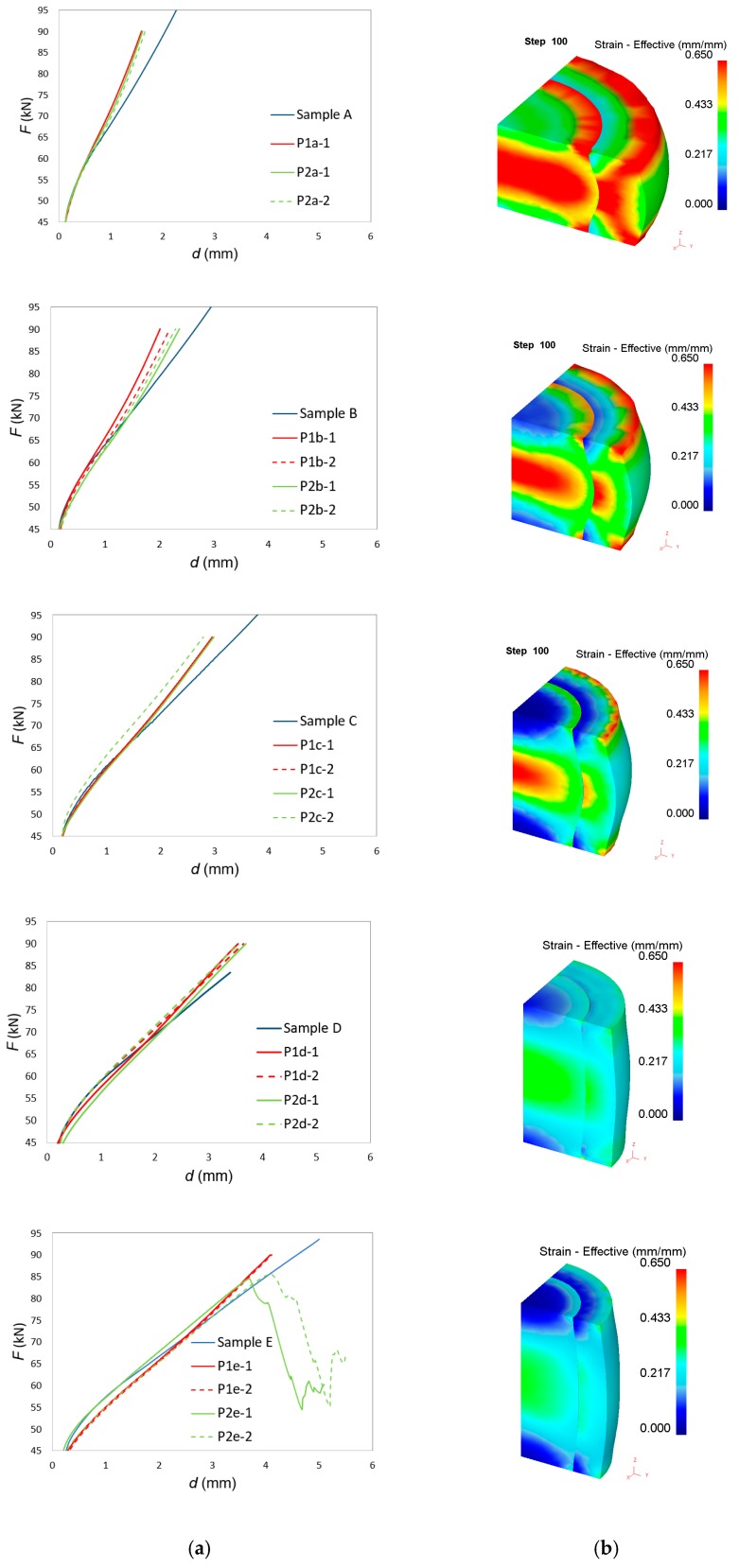
(**a**) Experimental and finite element predicted evolution of force with displacement for the entire set of test cases included in Table 4; (**b**) finite element predicted evolution of effective strain after 3.5 mm displacement of the upper die platen.

Figure 7a shows a comparison between the experimental and finite element predicted force-displacement evolution for the entire set of test samples included in Table 4.

The first conclusion to be taken from these results is the lack of influence of the type of assembly fit on the experimental evolution of the force with displacement, as it had been previously observed in finite elements.

The second conclusion is that the reason why finite elements are not able to predict the drop in force after cracking of the two test samples P2e is because modelling did not take crack propagation into consideration.

The third conclusion is that the overall agreement between experimental and numerical prediction of the force-displacement evolution improves as the shape factor H_0_/d_0_ increases. In the compression of single-material (solid) cylinders, this discrepancy was attributed to the influence of friction, which becomes more important and leads to more significant deviations, as the cylinders reduce their height—typically when the shape factor goes below 0.5 [26,27].

This type of influence was also observed in the compression of bimetallic cylinders, especially for test samples P1a-P2a and P1b-P2b with the lowest shape factors. Improvements of the numerical estimates would require tuning the friction factor for each shape factor H_0_/d_0_ in order to match the experimental results. This was not carried out because the actual differences between numerical and experimental results were considered not relevant for the overall aims and objective of the investigation.

The finite element distribution of effective strain after 3.5 mm displacement of the upper die platen is shown in Figure 7b. Effective strain values were obtained at the center of each tetrahedral element and interpolated between old (distorted) and new meshes during remeshing procedures [28]. As seen, the effective strain values are higher for small shape factors H_0_/d_0_ and the distribution is more homogeneous for high shape factors H_0_/d_0_. This result is interesting because it goes against the expected conclusion that cracks would be triggered in test sample P2e because its overall level of effective strain and its overall level of inhomogeneity would be the highest.

### 3.2. Ductile Damage

**Figure 8:**
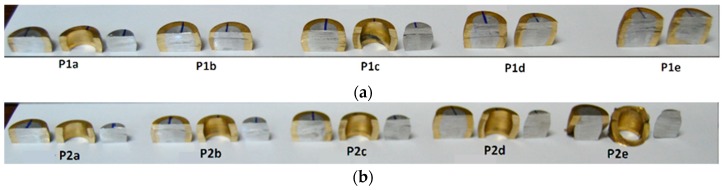
Cross section of bimetallic cylindrical test samples mounted with (**a**) interference fit (P1i) and (**b**) clearance fit (P2i), after compression.

Figure 8 shows the cylindrical test samples cut along their axial cross-sectional planes, after compression. As seen, some of the samples with clearance fit (P2i) showed a permanent joint between the center and the ring after the compression—one sample for each shape factor. On the contrary, two of the samples with interference fit (P1a and P1c) showed separation between the center and the ring after compression. The latter result was attributed to the appearance of internal voids at the contact interface, as reported by Cetintav et al. [1], who previously observed the existence of such voids for height reductions of 30%.

Because Essa et al. [17], also claimed the occurrence of voids when the ratio between the center and the ring diameters was higher than 0.6, it is not possible to claim a general design rule for obtaining bimetallic cylinders with permanent joints between the center and the ring after compression.

**Figure 9:**
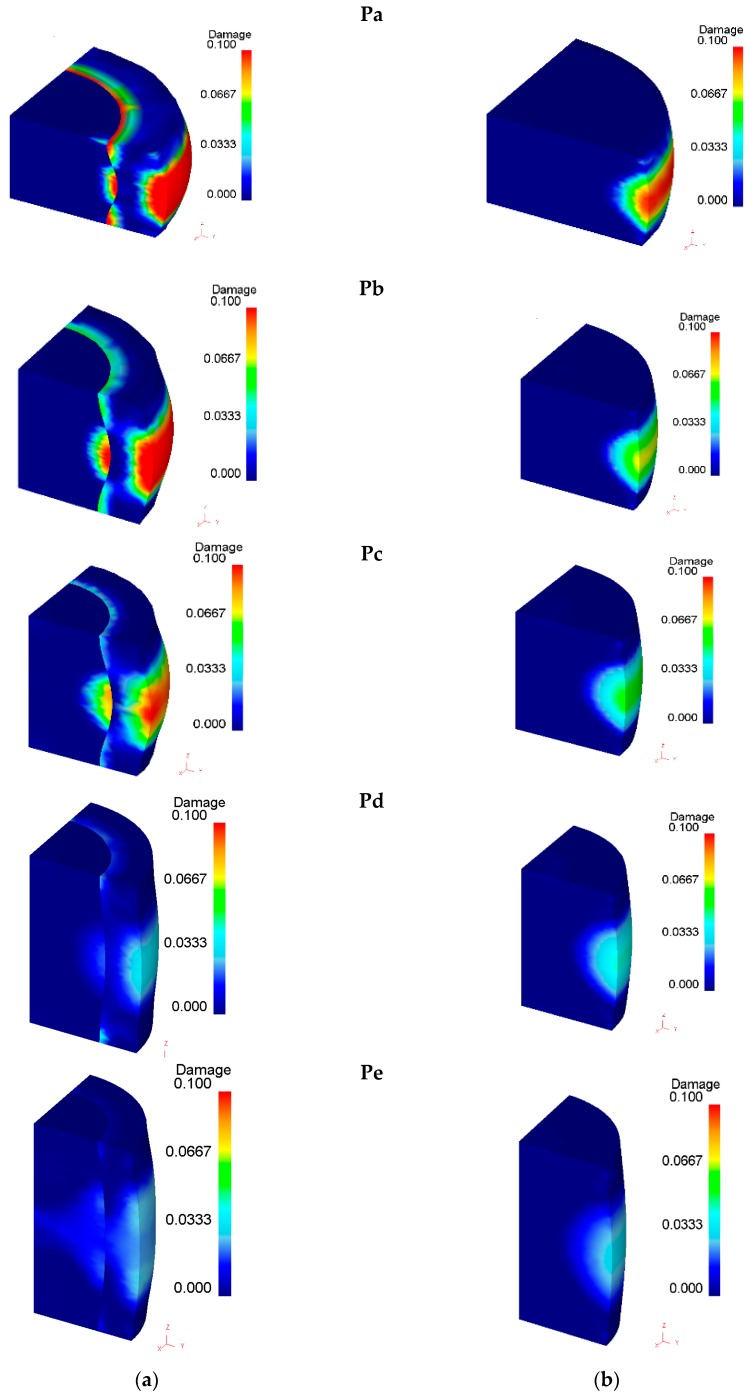
Finite element distribution of accumulated ductile damage in (**a**) bimetallic cylindrical test samples and (**b**) single-material cylindrical test samples made from the aluminum alloy UNS A92011 after 3.5 mm displacement of the upper die platen.

The distribution of accumulated ductile damage in both bimetallic and single-material cylinders made of the aluminum alloy UNS A92011 is disclosed in Figure 9. As seen, damage is higher in bimetallic cylinders and a discontinuity is also observed in the center-ring contact interface.

**Figure 10:**
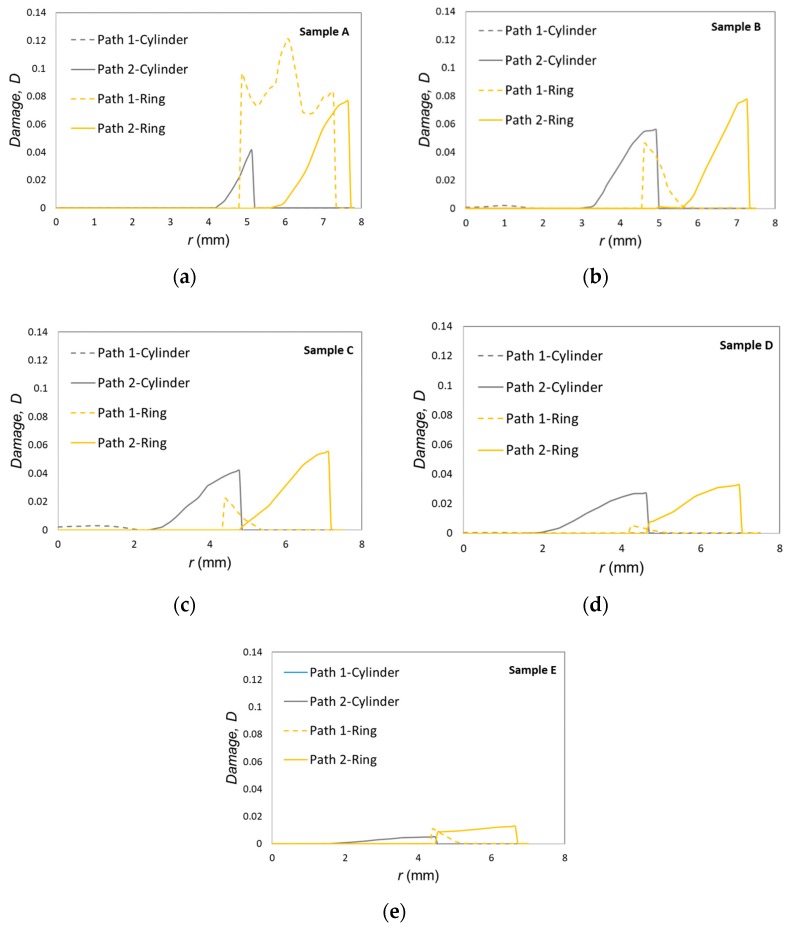
Finite element accumulated ductile damage as a function of the radial distance from the symmetry axis for paths 1 and 2 (Figure 4), after 3.5 mm displacement of the upper die platen: (**a**) Pa; (**b**) Pb; (**c**) Pc; (**d**) Pd; (**e**) Pe.

The accumulated ductile damage along paths 1 and 2 (Figure 3) is plotted in Figure 10. Results show that the higher the shape factor, the lower the damage in the center, especially for sample Pe, where damage is almost negligible due to the limited amount of inhomogeneous material flow (small amount of barreling).

Moreover, the mostly damaged region was found to occur at the middle plane of the outer ring surface, as previously claimed by Silva et al. [29]. The only exception is sample Pa, with the lowest shape factor, in which the highest value of damage, and therefore, the most critically damaged region, were also found at the intersection between the center-ring interface and the die platens (refer to path 1 in Figure 10). As expected, the cylinder center does not experience a significant amount of damage due to a nearly homogeneous material flow.

**Figure 11:**
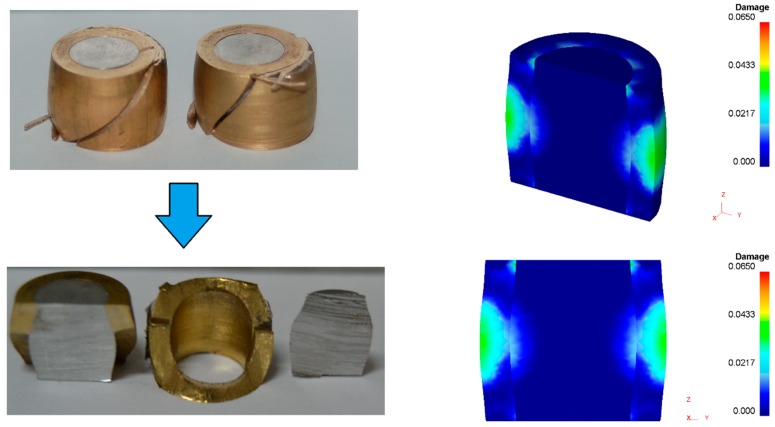
Cross section of the sample P2e showing failure by cracking and the corresponding finite element prediction of ductile damage (after 3.7 mm displacement of the upper die platen).

Now, focusing our attention of the test samples with the highest shape factor (samples Pe), one concludes that cracking in both samples P2e after a displacement of 3.7 mm is not compatible with the fact that the Pe samples are those presenting the smallest amounts of accumulated ductile damage. In fact, the finite element predicted damage was below 0.07 (Figure 11), and therefore, is more compatible with the absence of cracking observed in samples P1e than with the existence of cracks in samples P2e.

Despite the above-mentioned contradictory results, cracking in Figure 11 can only be explained by a combination between the maximum accumulated damage at the outer ring and at the intersection between the contact interface and the die platens. This explanation is not straightforwardly evident, but the experimental results allow concluding that the type of assembly fit (P1 versus P2) plays a key role on the development of cracks. In particular, interference fit is more favorable to preventing cracking of the ring.

### 3.3. Microstructural Observations

**Figure 12:**
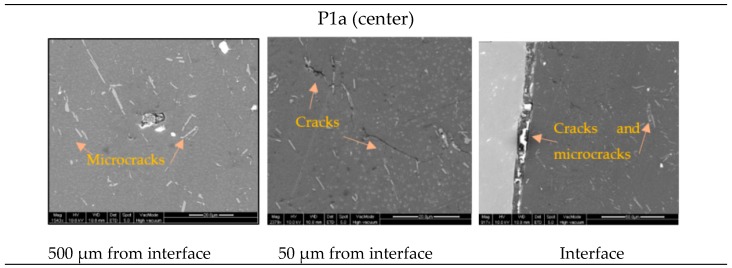

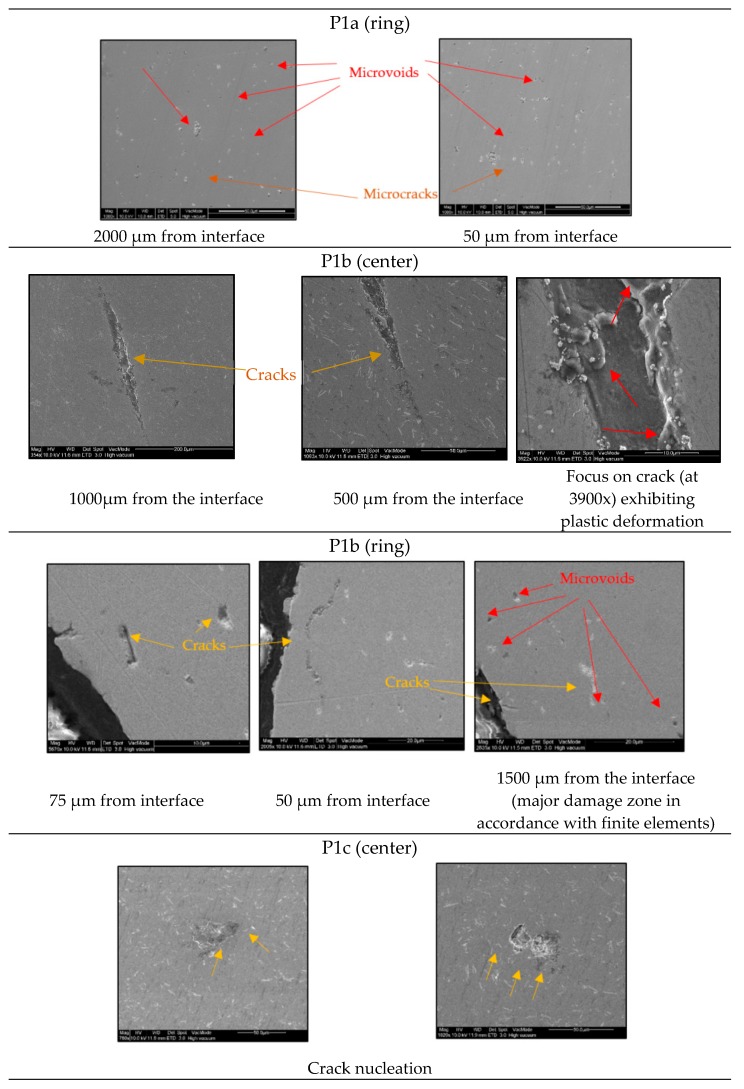

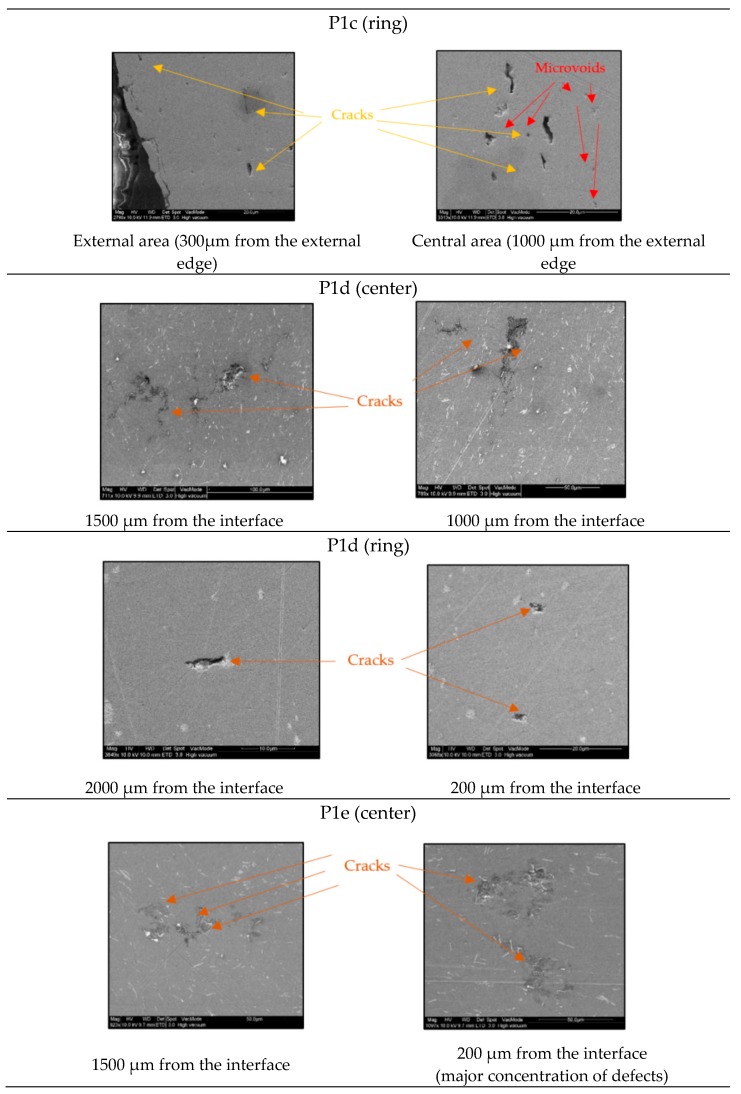
Microstructural observations in the center, and ring of the test samples’ interference fit (P1i).

Figure 12 shows different defects (voids, cracks, and microcracks) found in the center and ring in the tests samples with interference fit (P1i). The surface observed corresponds to the middle plane because it is the most damaged region, as described in Section 3.2.

Table 5 exhibits a comparison between the defects detected by SEM and the finite element predictions of ductile damage. The comparison is very good.

**Figure 13:**
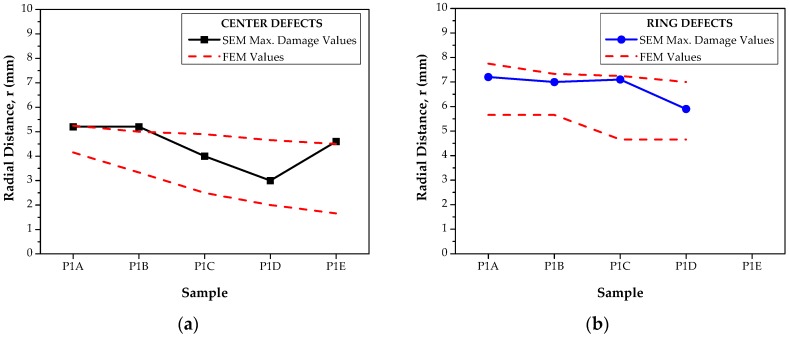
Representation of the locations with major presences of defects (maximum damage) observed by SEM, and the damage location range predicted by the finite element analysis. (**a**) Center; (**b**) ring.

Finally, Figure 13 shows, graphically, the locations of major presence of defects (maximum damage) observed by SEM and the damage location range predicted by the finite element analysis.

## 4. Conclusions and Future Work

This paper looked at the compression of bimetallic cylinders from a combined damage and microstructural point of view. The cylinders were made from an aluminum alloy UNS A92011 center and a brass UNS C38500 ring with various height to diameter ratios (“shape factor ratios”) and difference assembly fit tolerances (by interference and clearance). Ductile damage predictions were obtained from finite element modelling with a commercial finite element (FE) program, whereas the microstructural observations were carried out with a scanning electron microscope (SEM).

The comparison between the experimental and numerical predicted forces showed that the shape factor ratio influences the force-displacement evolution in a similar way to what is commonly found in the compression of single-material cylinders. Differences between experimental and numerical results were more significant for test samples P1a-P2a and P1b-P2b, having the lowest shape factors, due to variations in friction for large amounts of height reduction. In contrast, the type of assembly fit did not influence the overall force-displacement evolutions.

SEM observations of voids, microcracks, and cracks revealed a general good agreement with the finite element estimates of ductile damage. In particular, SEM observations detected voids, microcracks, and cracks in specific areas of the maximum predicted damage. Numerical simulations also showed that ductile damage is higher in bimetallic cylinders than in single-material cylinders made from the aluminum alloy UNS A92011, and that there is a discontinuity in ductile damage distribution at the contact interface between the center and the core. 

The overall results allow concluding that the higher the shape factor, the lower the damage, for the same amount of displacement of the upper die platen. This is due to differences in material flow inhomogeneity that favor the test samples with larger shape factors, but it is in clear contradiction to the fact that cracking was only found in test samples P2e having the largest shape factors. The explanation for this discrepancy was attributed to the type of assembly fit. In particular, results show that mounting the center in the ring with an interference fit prevents the occurrence of cracking. This is the most favorable condition for application in multi-material forming by compression. The amount of interference-fit needs to be addressed in future work, together with conducting complementary microstructural analysis in order to find relations between microstructure and material flow.

## Figures and Tables

**Table 1:** Main limitations in the fabrication of multi-material components by means of additive manufacturing, welding, and metal forming.

Main Limitations ^1^	Additive Manufacturing (DED and PBF)	Welding (Friction Stir Welding, Laser and Explosive Welding)	Metal Forming (Extrusion, Rolling, Upsetting)
Materials compatibility	X	X	-
Formation of brittle intermetalics	X	X	-
Microstructure thermal effects	X	X	-
Distortion	X	X	-
Residual stresses	X	X	-
Delamination	X	X	X
Formability limits	-	-	X

^1^ X: limitation associated to category of processes.

**Table 2:** Chemical compositions of the aluminum alloy UNS A92011 [22] and brass UNS C38500 [23].

Material	Al (wt.%)	Cu (wt.%)	Fe (wt.%)	Si (wt.%)	Zn (wt.%)	Pb (wt.%)
UNS A92011	92.0	5.5	0.7	0.4	-	-
UNS C38500	-	58.0	-	-	39.0	3.0

**Table 3:** Physical and mechanical properties of the aluminum alloy UNS A92011 [22] and brass UNS C38500 [23].

Property	UNS A92011	UNS C38500
Density (kg/m^3^)	2840	8470
Hardness (HB)	110	90–160
Youngs’ modulus (GPa)	70–72.5	90-100
Elongation A	6–12	15–25
Yield point (MPa)	125–230	220–350
UTS (MPa)	275–310	350–500

**Table 4:** Summary of the experimental work plan ^1,2^.

Group (Assembly Fit)	Sample	D_0_ (mm)	d_0_ (mm)	H_0_ (mm)	H_0_/d_0_
Interference	P1a	12	8	8	1.00
	P1b	12	8	10	1.25
	P1c	12	8	12	1.50
	P1d	12	8	14	1.75
	P1e	12	8	16	2.00
Clearance	P2a	12	8	8	1.00
	P2b	12	8	10	1.25
	P2c	12	8	12	1.50
	P2d	12	8	14	1.75
	P2e	12	8	16	2.00

^1^ The dimensional parameters (D_0_, d_0_, H_0_**)** are defined in Figure 1. ^2^ a,b,c,d,e: denotes the shape factor (H_0_/d_0_) of the sample.

**Table 5:** Comparison between SEM observations and finite element predictions of ductile damage.

Sample	SEM Observation	Finite Element Prediction of Ductile Damage
P1a	**Centre**: Concentration of defects (cracks, microcracks, microvoids) at 500 µm distance to the interface (*r* ≅ 5.20 mm) and in close agreement with the location of the maximum finite element prediction of damage (5.10 mm).	**Centre**: Accumulation of ductile damage for radial distances (*r*) between 4.15 and 5.25 mm (Figure 10). Maximum damage (*D* = 0.04) located at a radial distance of = 5.10 mm.
	**Ring**: Microcracks and microvoids located at 2000 µm distance from the interface (*r* ≅ 7.20 mm) and close to the maximum damage predicted by finite elements.	**Ring**: Accumulation of ductile damage for radial distances (*r*) between 5.66–7.75mm (Figure 10). Maximum damage (*D* = 0.078) located at a radial distance of 7.66 mm.
P1b	**Centre**: Concentration of defects (cracks, microcracks, microvoids) at 500–1000 µm distance to the interface (*r* ≅ 5.20mm) and in close agreement with the location of the maximum finite element predicted damage.	**Centre**: Accumulation of ductile damage for radial distances (*r*) between 3.33 and 5 mm (Figure 10). Maximum damage (*D* = 0.055) located at a radial distance between 4.66 and 4.90 mm.
	**Ring**: Microcracks and microvoids located at 2000µm from the interface (r ≅7.00 mm) and close to the maximum damage predicted by finite elements.	**Ring**: Accumulation of ductile damage for radial distances (*r*) between 5.66 and 7.33 mm (Figure 10). Maximum damage (*D* = 0.078) located at a radial distance between 7.1 and 7.33 mm.
P1c	**Centre**: Concentration of microcracks in the central area (between *r* = 3 and 5 mm).	**Centre**: Accumulation of ductile damage for radial distances (*r*) between 2.50 and 4.90 mm (Figure 10). Maximum damage (*D* = 0.04) located at a radial distance of 4.70 mm.
	**Ring**: Concentration of microcracks and cracks at 300-1000 µm distance from the interface (*r* ≅ 7.10 mm).	**Ring**: Accumulation of ductile damage for radial distances (*r*) between 4.66-7.25 mm (Figure 10). Maximum damage (*D* = 0.055) located at a radial distance of 7 and 7.10 mm.
P1d	**Centre**: Cracks at 1000-2000 µm distance from the interface (*r* ≅ 2.50 mm–3.5 mm).	**Centre**: Accumulation of ductile damage for radial distances (*r*) between 2.00 and 4.66mm (Figure 12). Maximum damage (*D* = 0.03) at a radial distance of 4.66 mm.
	**Ring**: Microcracks at 200–2000 µm distance from the interface (*r* ≅ 5–6.80 mm).	**Ring**: Accumulation of ductile damage for radial distances (*r*) between 4.66-7 mm (Figure 10). Maximum damage (*D* = 0.03) located at a radial distance of 6.33 and 7 mm.
P1e	**Centre**: Microcracks observed at 200–1500 µm distance from the interface (*r* ≅ 4.60 mm).	**Centre**: Accumulation of ductile damage for radial distances (*r*) between 1.66 and 4.5mm (Figure 10). Maximum damage (*D* = 0.005) at a radial distance between 3.33 and 4.33mm.
	**Ring**: No relevant defects were observed.	**Ring**: Accumulation of ductile damage for radial distances (*r*) between 4.4 and 6.66mm (Figure 10). Maximum damage (*D* = 0.01) located at a radial distance of 6 and 6.66 mm.
